# Genetic and evolutionary divergence of harbour seals (*Phoca vitulina*) in Iliamna Lake, Alaska

**DOI:** 10.1098/rsbl.2024.0166

**Published:** 2024-10-16

**Authors:** Tatiana Ferrer, Peter Boveng, Donna D. W. Hauser, David Withrow, Vladimir Burkanov, Thomas P. Quinn, Greg O'Corry-Crowe

**Affiliations:** ^1^ Harbor Branch Oceanographic Institute, Florida Atlantic University, Fort Pierce, FL 34946, USA; ^2^ NOAA Fisheries, Alaska Fisheries Science Center, Seattle, WA 98115, USA; ^3^ International Arctic Research Center, University of Alaska Fairbanks, Fairbanks, AK 99775, USA; ^4^ North Pacific Wildlife Consulting, Seattle, WA 98115, USA; ^5^ School of Aquatic and Fishery Sciences, University of Washington, Seattle, WA 98195, USA

**Keywords:** genetics, evolution, divergences, *Phoca vitulina*, Iliamna Lake, seals

## Abstract

Freshwater populations of typically marine species present unique opportunities to investigate biodiversity, evolutionary divergence, and the adaptive potential and niche width of species. A few pinniped species have populations that reside solely in freshwater. The harbour seals inhabiting Iliamna Lake, Alaska constitute one such population. Their remoteness, however, has long hindered scientific inquiry. We used DNA from seal scat and tissue samples provided by Indigenous hunters to screen for mitochondrial DNA and microsatellite variation within Iliamna Lake and eight regions across the Pacific Ocean. The Iliamna seals (i) were substantially and significantly discrete from all other populations (* $\bar{x}$ F*_st-mtDNA_ = 0.544, *$\bar{x}$ Φ*_st_ *_-_*_mtDNA_ = 0.541, *$\bar{x}$ F*_st-microsatellites_ = 0.308), (ii) formed a discrete genetic cluster separate from all marine populations (modal ∆*k* = 2, PC1 = 14.8%), had (iii) less genetic diversity (Hd, *π*, *H*_exp_), and (iv) higher inbreeding (*F*) than marine populations. These findings are both striking and unexpected revealing that Iliamna seals have likely been on a separate evolutionary trajectory for some time and may represent a unique evolutionary legacy for the species. Attention must now be given to the selective processes driving evolutionary divergence from harbour seals in marine habitats and to ensuring the future of the Iliamna seal.

## 1. Introduction

**Figure 1.**
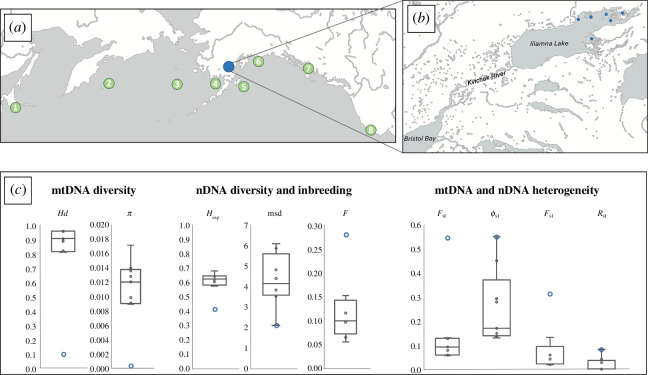
(*a*) Locations for eight harbour seal regions across the North Pacific range (green) and Iliamna Lake (blue). (*b*) Location of Iliamna Lake connected to Bristol Bay via the Kvichak River with sampling locations denoted by blue symbols. (*c*) Box-and-whisker plots (central tendency and variance) of genetic diversity, differentiation and inbreeding (see text for details). Iliamna seals are denoted by blue circles.

Few seal populations persist year-round in freshwater habitats. The small population of seals in Alaska’s Iliamna Lake (figure 1) is one of just five populations worldwide where seals are known to consistently inhabit freshwater lakes [1,2]. The other four are recognized as either distinct species (the Baikal seal, *Pusa siberica*), or subspecies (the Ladoga seal, *Pusa hispida ladogensis*, the Saimaa seal, *Pusa h. saimensis* and the Ungava seal, *Phoca vitulina mellonae*) [3,4]. The population from Iliamna Lake numbers approximately 400 individuals [5,6], has received comparatively little scientific attention to date [1,2,5,7,8], and provides an important cultural and dietary resource to local Indigenous communities [1,2]. Only relatively recently, a genetic study confirmed Iliamna Lake seals (termed ILS for the rest of this paper) were harbour seals, *Phoca vitulina*, and not the closely related spotted seal, *P. largha* [9].

Iliamna Lake is the largest in Alaska, with a surface area of 2622 km^2^, an average depth of 44 m and a maximum depth of 301 m [10], and it drains westward into Bristol Bay via the 110 km-long Kvichak River (figure 1*b*) providing the possibility for seals to move between the two water bodies and potentially interbreed. The lake supports a large population of sockeye salmon, *Oncorhynchus nerka*, that appears to provide a substantial, if seasonally limited, food resource [7]. Following a 2012 petition to list ILS as ‘Threatened’ or ‘Endangered’ under the US Endangered Species Act (ESA), the National Marine Fisheries Service (NMFS) conducted a status review. In 2016, NMFS concluded ILS were a ‘discrete’ population under the ESA but were unable to determine if they were also of ‘significance’ to the broader taxon, the eastern Pacific subspecies *P. v. richardii* [11,12]. NMFS’s determination of discreteness relied largely on a preliminary report indicating that ILS had low mitochondrial DNA (mtDNA) variation (all individuals possessed the same haplotype) and were likely genetically differentiated (for both nDNA and mtDNA) from seals in Bristol Bay, the closest marine habitat [9]. However, that study was constrained by small sample size (*n* = 13). Several questions remain that could significantly influence conservation and management actions on ILS, including the discreteness of this population and its significance to the species. In the current study, we substantially increased sample size from ILS through successful screening of seal scat samples for both mtDNA and nDNA (microsatellite) variations. We also assessed patterns of genetic diversity and levels of inbreeding within, and genetic differentiation between, ILS and eight regions of harbour seals across their Pacific range from Japan to California, encompassing the two recognized Pacific subspecies: the western *P. v. stejnegeri* and eastern *P. v. richardii*.

## 2. Methods

Samples were collected from six locations in Iliamna Lake between 1996 and 2017 (figure 1). DNA was extracted from 222 ILS scat and 13 tissue samples using the QIAamp^®^ Fast DNA Stool Mini Kit and the DNeasy^®^ Blood and Tissue purification kit (Qiagen), respectively. A 435 bp fragment of the mitochondrial control region and adjacent proline tRNA gene (mtDNA) was amplified and both forward and reverse strands were sequenced on a Genetic Analyzer 3130 (Applied Biosystems) according to published methods [13]. Twelve independent microsatellite loci, originally developed on pinnipeds and previously optimized for *P. vitulina* [14,15], were multiplexed and screened for genotypic variation: Hg3.6, Hg4.2, Hg6.1, Hg6.3, Hg8.9, Hg8.10, BG, SGPv9, SGPv11, Pvc19, Pvc78 and Lc28 [14,16–19] (electronic supplementary material, table S1). This 12-locus panel was successfully used to identify duplicate samples. The quality and quantity of DNA recovered from scat were much lower than from tissue samples, often resulting in poorer quality allele amplification and sequences. A rigorous QA/QC protocol was developed to ensure only high-quality genetic profiles were used in subsequent analyses. It comprised (i) high peak to noise requirements for allele and haplotype calls, and (ii) the same genotype scores from multiple replicate PCRs and screenings of the same individual.

Additionally, 205 samples chosen from eight regions across the Pacific, (based on earlier studies [13,20,21]), were sequenced for mtDNA and genotyped for 10 of the 12 microsatellite loci. The locations (1) Hokkaido, Japan, (2) the Commander Islands, Russia, (3) the Pribilof Islands, Alaska, (4) Bristol Bay, Alaska, (5) Kodiak, Alaska, (6) Prince William Sound, Alaska, (7) Southeast Alaska, and (8) Monterey Bay, California (figure 1*a*) span the ranges of the two currently recognized Pacific subspecies. The boundary between the western *P. v. stejnegeri* and eastern *P. v. richardii* is presumed to occur somewhere along the Commander-Aleutian ridge (i.e. between locations 2 and 3, figure 1*a*); however, no clear boundary has been established [20,21].

Minimum spanning networks of mtDNA haplotypes were generated using PopART [22] to reconstruct the phylogenetic relationships among haplotypes and their geographic distribution. Arlequin v. 3.5 [23] was used to estimate patterns of genetic diversity within geographic strata including frequency-based mtDNA haplotypic diversity (Hd), nDNA expected heterozygosity (*H*_exp_) and diversity indices that include information on the distance (i.e. number of mutations) among variants: mtDNA nucleotide diversity (*π*) and nDNA mean square distance (msd). Patterns of genetic differentiation among strata were estimated including frequency-based mtDNA and nDNA heterogeneity (*F*_st_) and their distance-based equivalents (*Φ*_st_ and *R*_st_, respectively). Homogeneity tests (comprising 50 000 iterations) were conducted to assess statistical significance in patterns of heterogeneity. Mega v. 6 [24] was used to determine pairwise differences among mtDNA haplotypes while Coancestry v. 1.01 [25] was used to calculate the triadic likelihood estimate (TrioML) of individual inbreeding coefficients (*F*) from the nDNA data. The Bayesian model-based clustering algorithm, Structure v. 2.3.4 [26], was used to infer the likely number of populations (*K*). Various parameter settings were evaluated, including whether the use of sampling location as a prior or admixture among population clusters was allowed or not, and each setting was run five times for each value of *K* to ensure convergence. For each parameter set, a burn-in period of 100 000 iterations was followed by 2 × 10^6^ iterations, and the modal value of the ad hoc statistic ∆*K* [27] determined the likely number of populations (*K*). Principal component analysis (PCA) on the genotypes of individuals was performed using the R package *adegenet* v. 2.1.10. Statistical outliers within the data were assessed relative to the upper and lower bound values. Swim distances among locations were measured as minimal distances across continental shelf waters and along the main course of the Kvichak River in Google Maps.

## 3. Results

Of the 222 scat samples analysed, 37 passed the internal QA/QC standards. Microsatellite genotyping identified four as duplicates, resulting in 33 scat samples and 13 tissue samples comprising the final Iliamna sample set. All 46 individuals were sucessfully genotyped, 41 of which were also successfully haplotyped (mtDNA). From the eight regions (figure 1*a*), 181/205 individuals were genotyped and all 205 individuals were haplotyped.

**Figure 2.**
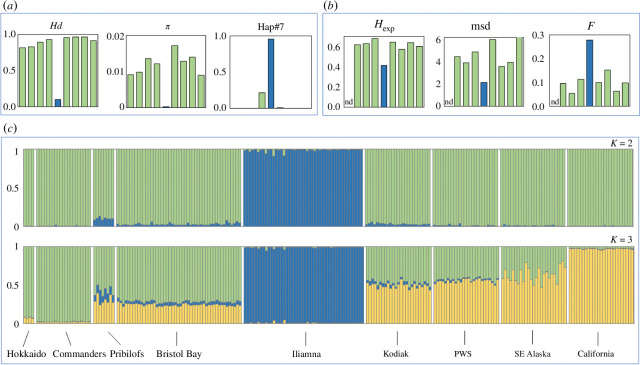
Patterns of genetic diversity and differentiation in Pacific harbour seals from eight marine regions (green) and Iliamna Lake (blue). (*a*) Patterns of mtDNA (haplotype) diversity within populations. (*b*) Patterns of nDNA (microsatellite) diversity and inbreeding within populations. (*c*) Summary plots of the model-based cluster analysis from structure for *k* = 2 and *k* = 3 population clusters. Both plots represent analyses where admixture was not allowed and sampling locations were not provided as prior information. All parameter settings yielded very similar results. Each of the 227 genotyped individuals is represented by a vertical bar with estimated membership of each cluster denoted by different colours. The order of the nine seal populations in each panel in A and B follow that of the plots in C. nd denotes no data.

**Figure 3.**
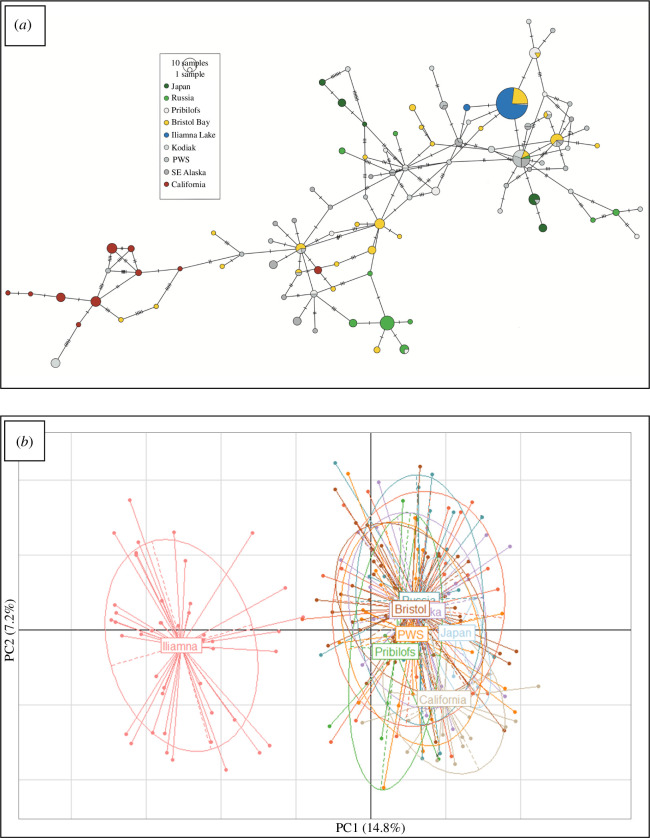
(*a*) Minimum spanning network of harbour seal mtDNA haplotypes from Iliamna lake and eight regions across the Pacific Ocean. For ease of viewing, the haplotypes found in Ilimana Lake are highlighted in blue and those in Bristol Bay in yellow. Samples from the geographical extremes are highlighted in shades of green (Japan and Russia) and in burgundy (California), while all others are in shades of grey. Disc size corresponds to overall haplotype frequency. (*b*) Principal component analysis on Pacific harbour seal genotypes from Iliamna Lake and eight marine regions. Individuals from a particular locale are represented by dots of the same colour and are connected to the locale’s labelled centroid. Confidence ellipses are included.

The level of mtDNA diversity (both Hd and *π*) within ILS was significantly lower than in all other regions (figure 1*c* and figure 2*a*); most ILS possessed haplotype #7 which was also the most common haplotype in Bristol Bay and was observed in Kodiak (figure 3*a*). Two ILS posessed a rare haplotype (#354), which has one substitution difference from haplotype #7 and from a haplotype found in PWS and SeAK (figure 3*a*). Of 1482 Pacific harbour seals analysed in our laboratory to date, this rare haplotype has only been detected in Iliamna Lake [28] (G. O’Corry-Crowe 2024, unpublished data). The level of nDNA diversity (both *H*_exp_ and msd) within ILS was also substantially lower than in all other regions (figures 1*c* and 2*b*). The estimated individual inbreeding coefficients (*F*) in ILS were, on average, significantly higher than in any of the other regions (figures 1*c* and 2*b*, electronic supplementary material, table S2). Low sample size prevented the inclusion of the Hokkaido population in these nDNA diversity analyses.

For mtDNA, ILS were significantly differentiated from seals in the other regions, including the nearest marine population, Bristol Bay (*p* < 0.0001, electronic supplementary material, table S3). Furthermore, the pairwise differences involving ILS ( $\bar{x}$ *F*_st_ = 0.544, $\bar{x}$ *Φ*_st_ = 0.541) were significantly higher than among the marine strata ( $\bar{x}$ *F*_st_ = 0.087, $\bar{x}$ *Φ*_st_ = 0.215), including the extreme ends of the Pacific range. For example, seals from Hokkaido, Japan were less different from California seals (8800 km away) than Bristol Bay seals were from the ILS population (110 km away).

Similarly, ILS were significantly differentiated for microsatellite markers from all the marine strata (*p* < 0.001, electronic supplementary material, table S3), and the pairwise differences involving ILS ( $\bar{x}$ *F*_st_ = 0.308, $\bar{x}$ *R*_st_ = 0.08) were substantially greater than differentiation among the marine locales ( $\bar{x}$ *F*_st_ = 0.043, $\bar{x}$ *R*_st_ = 0.011). The model-based cluster analysis revealed that Pacific harbour seals comprised two primary genetic clusters (modal ∆*k* = 2): (a) all marine locations and (b) Iliamna Lake (*K* = 2, figure 2*c*). The clusters were very discrete, with individuals having likelihoods >0.95 of originating in just one cluster. Furthermore, all parameter options assessed revealed the same two discrete clusters, including analyses allowing for admixture among strata and no sampling location as a prior. The next genetic break occurred among the marine strata with a clear distinction between the extreme western (Hokkaido and Commander Isles) and eastern (California) Pacific strata with intermediate locations having high likelihoods of shared ancestry from both clusters (*K* = 3, figure 2*c*). The PCA of individual genotypes also identified a primary genetic separation between ILS and all eight marine locales. The magnitude of the eigen value for PC1 far exceeded all others (0.514 versus ≤ 0.248), explained 14.83% of the total variation, and distinguished the freshwater seals from the marine seals (figure 3*b*).

## 4. Discussion

These findings are striking and unexpected. They not only run counter to hypotheses [1] that dispersal regularly occurs between the lake and marine habitats but indicate likely evolutionary, reproductive and demographic isolation of ILS from all Pacific Ocean populations, including the nearest marine population in Bristol Bay. The observed scales of genetic heterogeneity and low levels of genetic diversity recorded within ILS are consistent with historically small effective population size (*N*_e_) and strong genetic drift, likely indicating that ILS have been effectively isolated long enough for marked evolutionary divergence to occur. The genetic heterogeneity of ILS is especially striking when compared with the most geographically distant regions for the two Pacific subspecies, *P. v. richardii* and *P. v. stejnegeri*.

Caution is warranted when using contemporary patterns of genetic variation to reconstruct population histories, including estimating dispersal patterns and divergence times. The models linking dispersal rate and population size to genetic differentiation and diversity that underpin many genetic assessments of population structure and connectivity are based on idealized populations in drift–dispersal equilibrium [29,30]. Such conditions, however, are rarely met in nature [31]. Pinnipeds are no exception, often having complex demographic histories characterized by post-glacial expansions, source–sink dynamics, secondary contacts, founder events and bottlenecks. Additionally, different histories can theoretically produce similar patterns of genetic variation (e.g. [32]). For example, it should take a long period of effective isolation (e.g. thousands of years) for the levels of frequency-based differentiation (*F*_st_), we observed to arise via genetic drift among populations with large *N*_e_ and generation times like typical harbour seal populations. Alternatively, allele and haplotype frequencies could similarly diverge over much shorter time frames if one population was founded by individuals whose genetic make-up did not fully represent the source population (e.g. were close kin), and/or *N*_e_ was extremely low. In this scenario, the higher rate of genetic drift should also cause more rapid loss of genetic diversity.

However, our findings indicate that a recent origin of the ILS population seems unlikely. We found that all the marine populations, including Bristol Bay, had very high levels of mtDNA Hd (high Hd; figures 1*c* and 2*a*). Furthermore, a review of cases where multiple individuals (*n* = 5–12) within a marine population had been sampled at the same location on the same day revealed that harbour seals also tend to co-occur with others that typically have different haplotypes (G. O’Corry-Crowe 2024, unpublished data). It seems likely, therefore, that the original seals that founded the Iliamna Lake population, even if low in number, had multiple mtDNA lineages. To subsequently lose this diversity and drift to near fixation for a single haplotype (i.e. Hap#7) would likely have required a substantial amount of time. The lower distance-based (*π*, msd) estimates of mtDNA and nDNA diversity within ILS compared with other populations may provide further evidence that divergence was not recent where mutation as well as drift following a population split contributed to the geographic patterns of genetic variation detected today. Estimating population divergence times from genetic data alone relies on a series of assumptions [33] that often cannot be verified due to the complex interplay between behaviour, demography, drift and mutation at this taxonomic level. Dating founding events using geological history as, for example, the timing of the (re)emergence of suitable habitats and routes of colonization during successive interglacial periods, may be a more fruitful approach [4,21]. The origin of the Iliamna Lake population may date to when the lake was reachable and habitable sometime during the Holocene after the lake’s formation towards the end of the last ice age [34]. Indigenous knowledge is also informative where long-established oral traditions, that may span multiple centuries, do not recall a time when seals were not present in the lake while the first written record dates to Russian explorer Petr Korsakovskiy’s account of a seal hunt in 1819 [1]. More extensive sampling as well as the analysis of historical samples will help resolve the evolutionary history of the ILS further.

The high nDNA heterogeneity between Iliamna Lake and Bristol Bay, the very low likelihood of shared ancestry in all individuals from both populations (i.e. structure analysis) and the higher level of inbreeding within ILS compared with all marine populations indicate little or no recent gene flow into the Lake population. We cannot, however, exclude occasional movements of individual seals between the freshwater and marine habitats, and we note that any dispersal from Iliamna Lake would primarily contribute to the already relatively high frequency of Hap#7 in Bristol Bay.

This study also sheds new light on genetic structure across the Pacific. While the populations at the geographic extremes are markedly different (e.g. figures 2 and 3) and may still justify recognition as separate subspecies, our findings are also consistent with earlier studies [20] that a single polytypic form of harbour seal may exist in the Pacific Ocean with *stejnegeri* and *richardii* forming two extremes of a trans-Pacific cline. These findings highlight the need to fill remaining sampling gaps across the Aleutian-Commander ridge, and caution against relying on samples from the geographic extremes of continuously distributed species to reconstruct phylogenies and make taxonomic classifications. A recent genomics study of species-wide population structure also found marked differences between harbour seals in Japan and seals in the Gulf of Alaska and California but also noted the need to fill critical sampling gaps [21].

These findings will assist co-management and conservation efforts on ILS and should be viewed in concert with (a) the physical separation of ILS from other harbour seal populations, (b) their freshwater habitat and ecology [7,8] and (c) local Indigenous knowledge [1]. The ILS population has likely been on a separate trajectory for some time, and these analyses will help assess the population’s significance to the evolutionary legacy and potential of the species. As their numbers were likely never very large, they also offer a rare opportunity to investigate long-term survival and viability of small, isolated populations of slow reproducing large mammal species. In recent decades, Iliamna Lake has seen climate-driven increases in air and water temperatures, reductions in ice coverage [35] and lake level differences (T. P. Quinn 2023, unpublished data). Notably, these changes may affect seals directly or indirectly via factors such as sockeye salmon spawning [7].

The likely evolutionary, ecological, reproductive and demographic discreteness of ILS compared with other harbour seal populations indicate that they could be a unique endemic form of harbour seal. It remains unclear as yet whether this rises to the level of a unique subspecies as in other lacustrine seal populations. The Ungava seal, *P. v. mellonae*, found in Lac des Loups Marins, Nunavik, Canada, is the only other known freshwater harbour seal population. Their subspecies designation was based primarily on morphological differences, apparent isolation and some evidence for behavioural and ecological variations and haplotype divergence [36–38]. Revisions of the taxonomic status of ILS would be premature at this time. Future work should comprise morphological studies, including systematic craniometrics and pelage pattern analyses, and additional studies on behaviour and ecology. Genetic studies should also be expanded to include genomics and increase sample size. Genomics has recently proven highly informative in population structure analyses of harbour seals [21,39] but also offers tremendous potential in investigating selection, evolutionary potential and epigenetic adaptations.

The low levels of neutral diversity found within the ILS may indicate the population was never large. Their neutral diversity is lower than or comparable to other lacustrine seals, including subspecies that have witnessed recent population bottlenecks [4,40,41]. More research is required to further resolve the demographic history of ILS, including whether they experienced a bottleneck(s) in the past, and to assess the risk of future diversity loss and how that may influence population viability. Attention must also be given to the selective processes driving evolutionary divergence between freshwater habitats and their marine counterparts, to considering climate-related impacts, and ensuring the future of the Iliamna seal. The Iliamna seals also offer another system to study convergent adaptation of a number of populations of seal to freshwater.

## Data Availability

Editors and reviewers can access raw data from GenBank and by the Dryad link. Editors and reviewers are asked to abide by the authors' requirements on the use of these data. For reviewers: [42]. Tables are available in the electronic supplementary material and the data are made available on the Dryad Digital Repository [42,43].
